# Reliability in the Parameterization of the Functional Reach Test in Elderly Stroke Patients: A Pilot Study

**DOI:** 10.1155/2014/637671

**Published:** 2014-04-29

**Authors:** Jose Antonio Merchán-Baeza, Manuel González-Sánchez, Antonio Ignacio Cuesta-Vargas

**Affiliations:** ^1^Departamento de Psiquiatría y Fisioterapia, Instituto de Investigación Biomédica de Málaga (IBIMA), Universidad de Málaga, 29071 Málaga, Spain; ^2^School of Clinical Sciences, The Queensland University of Technology, Brisbane, QLD 4059, Australia

## Abstract

*Background*. Postural instability is one of the major complications found in stroke survivors. Parameterising the functional reach test (FRT) could be useful in clinical practice and basic research. *Objectives*. To analyse the reliability, sensitivity, and specificity in the FRT parameterisation using inertial sensors for recording kinematic variables in patients who have suffered a stroke. *Design*. Cross-sectional study. While performing FRT, two inertial sensors were placed on the patient's back (lumbar and trunk). *Participants*. Five subjects over 65 who suffer from a stroke. *Measurements*. FRT measures, lumbosacral/thoracic maximum angular displacement, maximum time of lumbosacral/thoracic angular displacement, time return initial position, and total time. Speed and acceleration of the movements were calculated indirectly. *Results*. FRT measure is  12.75 ± 2.06 cm. Intrasubject reliability values range from 0.829 (time to return initial position (lumbar sensor)) to 0.891 (lumbosacral maximum angular displacement). Intersubject reliability values range from 0.821 (time to return initial position (lumbar sensor)) to 0.883 (lumbosacral maximum angular displacement). FRT's reliability was 0.987 (0.983–0.992) and 0.983 (0.979–0.989) intersubject and intrasubject, respectively. *Conclusion*. The main conclusion could be that the inertial sensors are a tool with excellent reliability and validity in the parameterization of the FRT in people who have had a stroke.

## 1. Background

Stroke is the third leading cause of death and the leading cause of long-term neurological disability in the world [1, 2]. In Europe there are 250 strokes per 100,000 people each year, and this trend is worsening with time [2, 3]. Patients who survive a stroke often suffer a severe disability that causes major limitations in activities of daily living [2].

Postural instability is one of the major complications found in people who survive a stroke [2]; between 50% and 70% of patients who return to their homes from hospital or a rehabilitation centre experience falls [4]. In addition, a high percentage of patients experience greater difficulty to stand up, postural exaggeration, constant rebalancing in the sagittal and frontal plane, reduction of ability to be supported on the affected limb, and, therefore, an increased risk of falling [2, 5].

The instrumentalisation or parameterisation of functional test is to analyse the development of them acquiring parameters that can be used in clinical practice and basic research [6]. The use of standardised instruments measuring the health status of patients has been promoted in all fields of medicine applied to help establish and implement effective treatment strategies [7]. Because of their portability, reliability, and size, inertial sensors are instruments able to acquire kinematic variables of any gesture or movement [8].

In basic research, several studies have used inertial sensors to analyse the different kinematic variables that the gait can be decomposed [6, 8–11]. In clinical practice, this instrument has been used as a feedback tool for improving the sway on balance and ambulation tests [8].

The functional reach test (FRT) [12–16] is a clinically accepted tool to measure the semistatic balance of a subject because of its simplicity, reliability, economy, and portability. It is based on analysing the limits of stability in the absence of external shocks, assessing the maximum displacement, intentionally, which can reach a subject without losing balance. Thus, it integrates biomechanics, postural control, and subjective perceptions and correlates results of the greater chance of falling. This tool has been used to analyse the balance in patients suffering from Parkinson's disease, physical frailty, vestibular dysfunction, and stroke [17].

There are no studies in which FRT was instrumentalised by the inertial sensors in patients who have had a stroke.

The aim of this study is to analyze the reliability and validity of the parameterisation of FRT by using inertial sensors to record kinematic variables in subjects who have had a stroke. Our hypothesis is that the IS will be reliable instruments for kinematic study of the FRT.

## 2. Method

### 2.1. Design and Participants

In the cross-sectional study, participants met the following inclusion criteria: stroke verified as defined by the World Health Organization [18], independence walking for 10 m without the need to use physical support or the support of an auxiliary person, at a velocity equal to or less than 0.8 m/s, and the ability to remain standing with or without assistance for more than 30 seconds. People excluded from the study had experienced the following exclusion criteria: less than 65 years, with cardiovascular, respiratory, orthopedic, or severe metabolic problem, limitations in ambulation, serious problems of communication or understanding, history of a secondary neuronal pathology, and not giving informed consent.

Ethical approval for the study was granted by the ethics committee of the Faculty of Health Sciences, University of Málaga. This study was conducted in Accordance with Ethical Principles for Medical Research Involving Human Subjects (Helsinki declaration 2008).

Before performing the functional reach test, the information sheet and the informed consent were presented to each participant, in which the course of the study was explained. They were informed too about the voluntary nature thereof and the facility to leave the study at the moment they wanted, as well as the protection of their personal data according to the Organic Law of Protection of Data Personal 19/55.

### 2.2. Inertial Sensors

The inertial sensors used in this study were the model InertiaCube3TM InterSense Inc. (Bedford, MA, USA) with a sampling frequency of 180 Hz.

The InertiaCube3 is a sensor based on microelectro-mechanical systems (MEMS) technology without involving casters, which could generate noise, inertia forces, and mechanical failures. The InertiaCube measures nine simultaneously physical properties, that is to say, angular rates, linear accelerations, and magnetic field components along the three axes (Yaw, Pitch, and Roll). Miniature vibrating elements are used for measuring all the components of the angular velocity and linear accelerations.

### 2.3. Functional Reach Test (FRT)

The subjects were standing, parallel to a wall, close to but not touching, and with their feet open to shoulder height. The shoulders were positioned at 90 degrees of flexion, with elbows and hands extended. In this position, on a yardstick, the assessor recorded, at the third metacarpal head, the starting position. The subject held this position for three seconds. Then, without moving their feet off the ground, the participant performed hip flexion by moving their trunk forward and reaching as far as they could without taking a step. At this point, the assessor located the position of the third metacarpal. Subsequently, the participant returned to the starting position and remained still for a further three seconds to clearly differentiate the end of the movement. The difference in centimeters between the first and second mark during the functional reach test was FRT value.

The FRT was performed three times, but the average of the last two was considered the FRT measure.

Before starting, it was explained to the participants the movement execution. The subjects could take all the tests considered necessary for better understanding of the test, which has a reliability of 0.81 [19].

**Figure 1:**
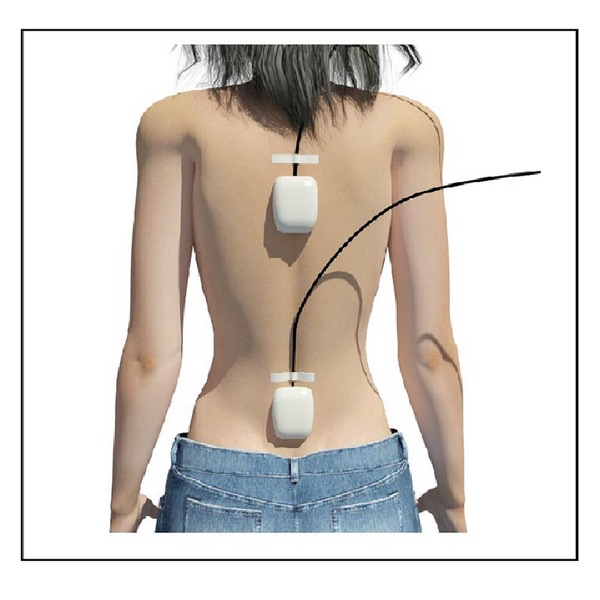
Position of the inertial sensors on the back of patients.

Two inertial sensors were placed, one in the centre of mass and the other in the trunk (Figure 1), which made a cinematic record during test execution. Registration of the kinematic variables of test development was carried out throughout the test over the initial and final three seconds. This served the subject to reach the starting position and the researcher as a reference to analyze the data. The analysis was performed with performance that had greater distance in the FRT.

**Figure 2:**
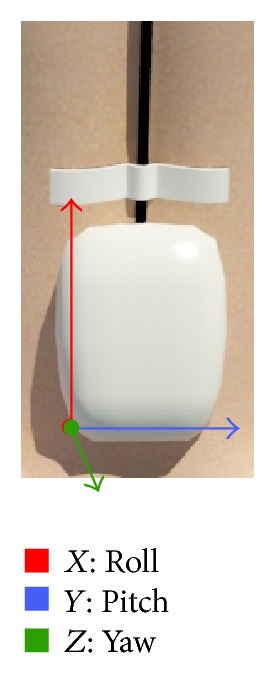
Origin of the coordinates (*X*, *Y*, *Z*) in the inertial sensor.

The sensor was placed so that the origin of the coordinates (*X*, *Y*, *Z*)  (0,0, 0) was positioned in the posteroinferior left corner (Figure 2).

After completion of data collection, a blinded investigator performed offline extraction of variables from each of the graphs generated following completion of each test.

### 2.4. Outcome Measures

The outcomes measures extracted from FRT or Duncan test were as follows:* FRT distance*: the distance in centimeters that the subject is able to achieve during the realization of the FRT;* maximum angular lumbosacral/thoracic displacement FRT*: the angular variation on the pitch in the subject during the performance of FRT axis; this amplitude is considered from the time the test begins until peaking imbalance before returning to the starting position;* time maximum angular lumbosacral/thoracic displacement FRT*: the time that the subject takes to reach the peak during running the FRT;* time return starting position*: the time that the subject takes to return to a starting position from reaching the peak;* total time FRT*: the time the subject takes from start to perform the FRT until he returns to his starting position. All variables listed above were extracted from the registry of the inertial sensor in the pitch axis.

Subsequently, using the extracted data, the following variables were calculated:* average speed FRT*: average rate at which the subject performs all the FRT;* maximum angular lumbosacral/thoracic displacement speed FRT*: the average velocity at which the subject reaches the peak from the start of carrying out the FRT;* starting to return position speed*: the average rate at which the subject performs the movement back to the starting position to the maximum peak;* average acceleration FRT*: the mean acceleration at which the subject carries out the FRT;* maximum angular lumbosacral/thoracic displacement average acceleration FRT*: the average acceleration at which the subject develops the test from the beginning until he reaches the peak;* acceleration average return starting position FRT*: the average acceleration of the subject from reaching the peak until the return to his starting position.

In addition, the* mean and standard deviation of X*, *Y*, and *Z* were calculated in the highest, lowest, and average speed and acceleration in both sensors, just as the* mean and standard deviation in the resultants* of displacement and resultants of minimum and maximum speed and acceleration. Resultant is calculated previously by finding the square root of the sum of the squares of the three axes in movement, the maximum and minimum of speed and acceleration of FRT.

The outcome analyzed was the highest value obtained during the performance of the three repetitions of the test.

### 2.5. Procedure

Before beginning, the test was explained in detail to participants and the participants signed the informed consent. Sociodemographic data and anthropometric measures of each subject were collected. To improve the description of the sample, participants completed the Barthel Index (BI), the Stroke Impact Scale-16 (SIS-16), and the Canadian Neurological Scale (CNS). The reliability of these tools is Kappa = 0.93 [20, 21], Kappa = 0.76 [22], and ICC = 0.70 to 0.92 [23], respectively.

**Figure 3:**
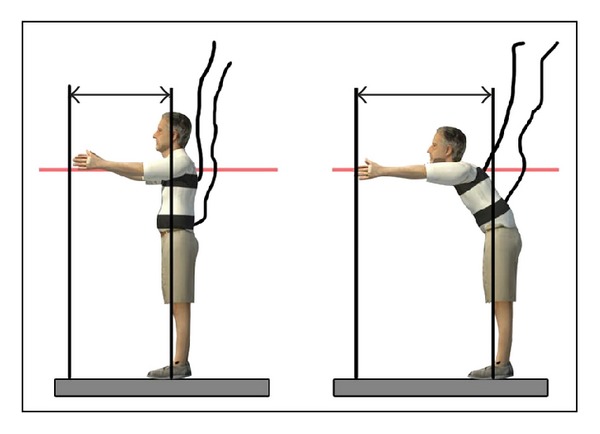
Performing of FRT.

Functional reach test (FRT) or Duncan's test (Duncan 1990) was performed. During execution, the subjects carried two inertial sensors, one was placed at the level of L_5_-S_1_ (lumbar) and the other in T_7_ (trunk) (Figure 3). Two researchers monitored the implementation of the test and performed the analysis of the results independently. Under the supervision of individual researchers, the test was performed three times, considering the average of the last two repetitions the measure of the FRT.

After analyzing the data obtained in the kinematic registration by inertial sensors, a number of direct and indirect variables were obtained. Direct variables obtained were time and displacement between each of the points of the three intervals. And the indirect variables, calculated thereafter, were the speed, acceleration, and the resultant.

### 2.6. Data Analysis

After completing the sample, a descriptive analysis was made, which included anthropometric measurements and the results of various self-administered questionnaires specifically designed for patients with neurological affectations. A descriptive analysis of all kinematic outcomes recorded by the two inertial sensors (trunk and lumbar) was developed and the average range achieved in the FRT.

After performing the normality of the variables by Kolmogov-Smirnov (KS) test, the results were compared, records between trunk and lumbar, both directly measured outcomes (time and displacement) and outcomes obtained indirectly (velocity, acceleration, and resultant). For parametric outcomes, Student's *t*-test was used, and for the nonparametric, Wilcoxon's test was used. The index of significance was established in less or equal to *P* = 0.05 values.

Reliability measures were calculated by analysing the internal consistency (intraclass correlation coefficients were calculated for intrarater and interrater reliability) of the measures with 95% confidence interval of each outcome variable. The reliability was calculated in the functional reach test and the outcomes measured by the IS (time and displacement). The reliability of the indirect variables was not calculated (velocity and resultant acceleration), because its value is determined by the reliability of the direct measures. The levels of reliability were excellent (ICC > 0.80), good (0.80 > ICC > 0.60), moderate (0.60 > ICC > 0.40), and poor reliability (ICC < 0.40) [24].

The Statistical Package for the Social Sciences (SPSS) (version 17.0 for Windows, Illinois, USA) was used to represent the statistical analysis.

## 3. Results

Table 1 shows the anthropometric and demographic data of the participants, as well as the values of the various specific tests that each participant completed as well as the values of the various specific tests which were intended to identify the degree of involvement of the patient as a result of the stroke.

Table 2 presents the description of the kinematic variables of the FRT based on their placement in the centre of mass and thorax, distance functional reach test, and the number of participants. Three intervals of movement based on the following points were considered: beginning of the test, maximum angular displacement, and end of the test. The outcomes calculated in each of these intervals were time, displacement, velocity, and acceleration. It can be seen in Table 2 the maximum, minimum, mean, and standard deviation of each outcome.

Table 3 shows the resultant displacement, resultant in the maximum and minimum velocity, and the resultant in maximum and minimum acceleration in the FRT, and minimum of speed and acceleration. All the outcomes previously mentioned have been presented as mean and standard deviation of the sum of the participants relating to *X*, *Y*, *Z* of each of the sensors and the difference between them.

Table 4 presents the intrasubject and intersubject reliability of the outcomes measured directly in the parameterisation of the FRT. Intrasubject reliability values observed in the use of inertial sensors are all located above 0.820, ranging from 0.829 (time B_C lumbar area) to 0.891 (A_B displacement of the trunk). Likewise, the observed intersubject values range from 0.821 (time B_C lumbar area) to 0.883 (B_C trunk displacement). On the other hand, the reliability of the FRT was 0.987 (0.983–0.992) and 0.983 (0.979–0.989) intersubject and intrasubject, respectively.

## 4. Discussion

After analyzing the data obtained, it shows how the inertial sensors are a reliable, specific tool for the parameterization of a functional reach test in a sample of stroke patients who suffer from problems of imbalance, and we can say that the aim of this study was achieved. Furthermore, based on the results, the hypothesis set out at the beginning is confirmed.

No study was found that uses inertial sensors to parameterize the FRT. However, these instruments have been used themselves for the kinematic analysis of other tests [6, 25–31], and these were static [27, 31], semistatic [28, 29], or dynamic [6, 25, 26].

Reliability levels observed in the present study could be categorized as excellent [24] in base of the results that the intraobserver reliability ranges between 0.829 and 0.878 and the interobserver between 0.821 and 0.883 (Table 4). These results are in accordance with all the studies consulted [6, 25, 27–31], except the study of Lugade et al. [26], which showed reliability levels over 0.9.

These results are consistent even if we consider some details of registration, such as the position of the sensor, where the values of obtained reliability (ICC: 0.835–0.877 (trunk) and 0.829–0.878 (lumbar)) are comparable with other studies that share the sensor location, as in the study of Kavanagh et al. [25], who analyzed the reliability of IS to analyze the progress at different speeds (slow, determined by the participant, and fast), placing, among others, IS in trunk and lumbar, which achieved a reliability of 0.83–0.93 (trunk) and 0.78–0.92 (lumbar) during performance on the speed determined by the participant.

Considering the observer test, an intraobserver reliability of 0.829–0.878 and interobserver of 0.821–0.883 (Table 4) were noted, which are similar to those reported by Kavanagh et al. [25]: ICC values (95% IC) of 0.84–0.91 (intraobserver) and from 0.85 to 0.93 (interobserver).

The reliability of runtime testing, total or different partials, demonstrated excellent intrasubject reliability with ICC values (95% IC) of 0.863–0.877 (trunk) and 0.829–0.867 (lumbar) (Table 4). These values are comparable with those presented by Duffy et al. [21] in the other semistatic test (sit to stand), where the reliability values were 0.89 (0.78–0.94), 0.83 (0.67, 0.92), and 0.8 (0.61, 0.9) for the total time of the test, stand to sit time, and sit to stand time, respectively. In addition, as regards the reliability of the time keeps in the inter-observer analysis with values ranging between 0.821 and 0.858, respectively.

In analyzing the data from each inertial sensors, it can be observed how the registered values of each sensor are very broad with respect to the standard deviations presented (Tables 2 and 3), in both the trunk and the lumbar. Moreover, the different registration observed between the lumbar and the trunk sensor (Table 3) confirms that the inertial sensors, in addition to being sensitive, are tools with high specificity. These results are consistent with other studies that have also found inertial sensors as instruments with high sensitivity and specificity [29, 31].

On the other hand, in analyzing the reliability of the measures of the functional reach, it is observed, with regard to people who have suffered a stroke, that the reliability levels are greater than 0.98 (ICC: 0.987 (0.983–0.992) and 0.983 (0.979–0.989) for intra- and interobserver). These reliability levels are not consistent with those observed in previous studies, where FRT reliability levels were 0.86 [15] and 0.64–0.74 [32]. The difference between levels of reliability can be because, in the present study, participants were stroke victims, which determine the imbalance in the functional reach (12.75 (11–15) cm), thus limiting, in turn, the variability of the measuring and improving the reliability. However, in other studies consulted, the study subjects are patients suffering from Parkinson's disease [32] or are healthy older women [15]. These participants achieved values in FRT in excess of those obtained in the present study: 33.54 (±7.36) [32] and 17.1 (±6.7) [15] FRT.

Age also appears to be a negative determinant of the results obtained in the FRT. Several studies on stroke patients have been published and the results of functional reach are not comparable with those observed in the present study [12, 13], since in both cases the mean values observed in these studies are double (24.6–25.6 cm [13] and 28.0 cm [14]) those presented in Table 2 (12.75 cm ± 2.06). The difference may reside, as we indicated earlier, in the average age of participants, 56.3–56.8 years [12] and 55.9–56.3 years [14], respectively. However, when participants have a similar age, the results observed are consistent with the present study. The values presented by Palsbo et al. [33] and DeWaard et al. [15]—values in the FRT of 2.7–17.0 cm [33] and 17.1 (8.9–26.0) cm [15]—are similar to those obtained in the present study (2.06 ± 12.75 cm); the mean age in each of the studies was 80.8 (66–90) years [15] and 64 [33], compared with 76.7 years in the present study.

The present study has strength in observing that the parameterization of FRT allows obtaining reliable and valid kinematic measures with a high potential for research in the clinical field, either in the assessment or the monitoring of different types of patients. However, it also has some weaknesses such as the lack of a control group or restriction on the right side as the affected side of the patient. In addition, the results presented in this study are those obtained in a pilot trial; however, it is necessary to expand the sample to obtain the results of sensitivity and specificity of inertial sensors in the parameterisation of FRT.

## 5. Conclusions

The main conclusion that can be reached is that the inertial sensors are a tool with excellent reliability, validity, sensitivity, and specificity in the parameterisation of the functional reach test in individuals who have had a stroke.

## Figures and Tables

**Table 1:** Descriptive values of participants.

	Minimum	Maximum	Mean	SD
Age	69	92	76.7	

CNS	8.0	9.0	8.500	0.4082
BI	85	100	92.50	6.455
SIS_16	57	75	67.00	7.832

*N* valid (according to the list)	4			

CNS: Canadian Neurological Scale; BI: Barthel Index; SIS-16: Stroke Impact Scale-16.

**Table 2:** Description of the kinematic variables of FRT depending on the placement of the sensor.

		Minimum	Maximum	Mean	SD
Functional reach test distance		11	15	12.75	2.06

Trunk	Time A_B (s)	7.05	9.97	8.71	1.5
	Displacement A_B (°)	4.73	20.55	13	7.94
	Speed A_B (°/s)	0.67	2.06	1.49	5.29
	Acceleration A_B (°/s^2^)	0.10	0.21	0.17	3.53
	Time B_C (s)	2.09	12.98	6.96	5.53
	Displacement B_C (°)	5.16	12.88	9.8	4.1
	Speed B_C (°/s)	2.47	0.99	1.41	0.74
	Acceleration B_C (°/s^2^)	1.18	0.08	0.20	0.13
	Time A_C (s)	12.06	20.03	15.68	4.03
	Displacement A_C (°)	6.2	20.58	13.5	7.19
	Speed A_C (°/s)	0.51	1.03	0.86	1.78
	Acceleration A_C (°/s^2^)	0.04	0.05	0.05	0.44

Lumbar	Time A_B (s)	5.55	12.15	8.56	2.93
	Displacement A_B (°)	4.61	11.1	7.49	3.25
	Speed A_B (°/s)	0.83	0.91	0.88	1.11
	Acceleration A_B (°/s^2^)	0.15	0.08	0.10	0.38
	Time B_C (s)	2.38	13.37	8.13	6.019
	Displacement B_C (°)	6.62	14.93	9.74	3.68
	Speed B_C (°/s)	2.78	1.12	1.20	0.01
	Acceleration B_C (°/s^2^)	1.17	0.08	0.15	0.02
	Time A_C (s)	11.98	20.32	16.7	3.7
	Displacement A_C (°)	9.22	22.2	14.98	6.49
	Speed A_C (°/s)	0.77	1.09	0.89	1.75
	Acceleration A_C (°/s^2^)	0.06	0.05	0.05	0.47

*N* valid (according to the list)		4			

A: beginning of the FRT; B: maximum angular displacement; C: end of the FRT.

**Table 3:** Mean and standard deviation of the records of each of the sensors and differences between them.

	Trunk			Lumbar			Mean difference		
	*X*	*Y*	*Z*	*X*	*Y*	*Z*	*X*	*Y*	*Z*
Resultant displacement	**33.87**			**36.45**			**1.86**		
	(±6.71)			(±14.01)			(±23.64)		

Speed mean	**1.72** (±0.21)	**26.37** (±7.20)	**23.30** (±9.54)	**1.74** (±0.75)	**24.25** (±7.98)	**20.10** (±5.59)	**0.01** (±0.91)	**−2.32** (±2.83)	**−2.31** (±13.18)
Maximum speed	**−0.60** (±0.74)	**10.27** (±4.15)	**9.77** (±1.52)	**1.77** (±1.18)	**10.53** (±6.50)	**8.38** (±1.27)	2.00* (±0.75)	**0.64** (±5.13)	**−1.32** (±2.40)
Minimum speed	**−2.32** (±0.92)	**−16.10** (±3.10)	**−13.53** (±8.08)	**0.03** (±1.56)	**−13.72** (±4.99)	**−11.72** (±4.39)	**2.00** (±1.54)	**2.97** (±5.72)	**0.99** (±10.80)

Maximum resultant speed	**14.34**			**13.86**			**−0.12**		
	(±3.67)			(±5.89)			(±5.66)		
Minimum resultant speed	**21.74**			**18.53**			**−3.05**		
	(±6.19)			(±4.74)			(±3.29)		

Mean acceleration	**2.17** (±1.28)	**3.27** (±1.48)	**6.53** (±1.32)	**1.27** (±2.51)	**−0.54** (±4.50)	**4.75** (±1.01)	**−0.24** (±1.50)	**−3.96** (±4.53)	**−1.71** (±1.28)
Maximum acceleration	**−0.81** (±1.44)	**−2.90** (±3.07)	**95.40** (±8.54)	**−0.00** (±0.86)	**−2.16** **(±1.43)**	**92.11** **(±4.75)**	**1.04** (±1.95)	**0.81** (±4.57)	**−5.30** (±5.85)
Minimum acceleration	**−2.98** (±2.34)	**−6.17** (±3.47)	**88.88** (±9.58)	**−2.41** (±0.90)	**−1.62** (±4.54)	**87.36** (±4.66)	**0.66** (±2.66)	**4.77** (±3.46)	**−3.59** (±7.02)

Maximum resultant acceleration	**95.50**			**92.15**			**−5.36**		
	(±8.41)			(±4.76)			(±5.70)		
Minimum resultant acceleration	**89.22**			**87.50**			**−3.77**		
	(±9.36)			(±4.66)			(±6.69)		

Significance level: *P < 0.05.

**Table 4:** Intraobserver and interobserver reliability of variables measured directly during functional reach test.

			SEM (stand. error measu.)	Intraobserver			Interobserver		
	Variable			ICC	IC (95%)		ICC	IC (95%)	
					Min.	Max.		Min.	Max.
Trunk	Time	A_B	**0.867**	**0.855**	0.833	0.872	**0.851**	0.828	0.869
		B_C	**4.582**	**0.835**	0.822	0.852	**0.831**	0.824	0.848
		A_C	**3.194**	**0.847**	0.839	0.868	**0.840**	0.839	0.868
	Displacement	A_B	**2.364**	**0.891**	0.879	0.913	**0.883**	0.877	0.913
		B_C	**2.329**	**0.863**	0.843	0.878	**0.858**	0.845	0.871
		A_C	**4.153**	**0.877**	0.861	0.895	**0.870**	0.859	0.888

Lumbar	Time	A_B	**1.463**	**0.867**	0.844	0.880	**0.858**	0.841	0.879
		B_C	**1.624**	**0.829**	0.806	0.855	**0.821**	0.804	0.852
		A_C	**3.011**	**0.851**	0.837	0.869	**0.839**	0.832	0.860
	Displacement	A_B	**1.840**	**0.878**	0.850	0.896	**0.875**	0.852	0.893
		B_C	**1.851**	**0.868**	0.849	0.883	**0.863**	0.846	0.870
		A_C	**1.738**	**0.872**	0.853	0.889	**0.868**	0.850	0.877

Functional reach test				**0.987**	0.983	0.992	**0.983**	0.979	0.989
